# Efficiency and safety increases after the implementation of a multi‐institutional automated plan check tool at our institution

**DOI:** 10.1002/acm2.12845

**Published:** 2020-03-20

**Authors:** Sean L. Berry, Ying Zhou, Hai Pham, Sharif Elguindi, James G. Mechalakos, Margie Hunt

**Affiliations:** ^1^ Department of Medical Physics Memorial Sloan Kettering Cancer Center New York NY USA

**Keywords:** automation, plan checking, quality and safety, treatment planning

## Abstract

**Purpose:**

The plan check tool (PCT) is the result of a multi‐institutional collaboration to jointly develop a flexible automated plan checking framework designed with the versatility to be shared across collaborating facilities while supporting the individual differences between practices. We analyze the effect that PCT has had on the efficiency and effectiveness of initial chart checks at our institution.

**Methods and Materials:**

Data on errors identified during initial chart checks were acquired during two time periods: before the introduction of PCT in the clinic (6/24/2015 to 7/31/2015, 187 checks) and post‐clinical release (4/14/2016 to 5/2/2016, 186 checks). During each time period, human plan checkers were asked to record all issues that they either manually detected or that were detected by PCT as well as the amount of time, less breaks, or interruptions, it took to check each plan.

**Results:**

After the clinical release of PCT, there was a statistically significant decrease in the number of issues recorded by the human plan checkers both related to checks explicitly performed by PCT (13 vs 50, *P* < 0.001) and in issues identified overall (127 vs 200, *P* < 0.001). The mean and medium time for a plan check decreased by 20%.

**Conclusions:**

The use of a multi‐institutional, configurable, automated plan checking tool has resulted in both substantial gains in efficiency and moving error detection to earlier points in the planning process, decreasing their likelihood that they reach the patient. The sizeable startup effort needed to create this tool from scratch was mitigated by the sharing, and subsequent co‐development, of software code from a peer institution.

## 1. INTRODUCTION

Radiotherapy initial chart checks are a highly effective method of catching errors before they reach the patient but human lapses in detection are a limiting factor.^1^ It has been suggested that automating the checks would lead to higher rates of error detection^2^ and furthermore, would allow human planners and plan checkers to direct their focus away from the review of technical details and toward more thorough analysis of plan quality. Automation should also increase the efficiency of the process, which is currently very resource intensive. At the time of this analysis, on the main campus of our institution, 2.2 full‐time equivalent (FTE) physicist positions were devoted solely to initial chart checks. This duty is spread, on a rotating basis, over the most senior members of the treatment planning section, consisting of 10 FTEs.

Despite the potential benefits, the adoption of automation has reportedly been slow due to the large upfront effort, in terms of software development and testing, necessary before realizing gains on a chart to chart basis.^3^ There are a number of automated plan checking approaches described in the literature, the majority of which have been single institutional. These checkers compare data consistency between the treatment planning system (TPS) and the record and verify system (R&V)^4^, ^5^ and many also perform a limited set of integrity and consistency checks, such as calculation model and grid size, naming conventions, isocenter consistency, couch positions, and appropriateness of control points (CP) and monitor units (MU).^4^, ^5^, ^6^, ^7^, ^8^ While these checkers have demonstrated value to their individual departments, the value to the field at large is limited by their specificity to that clinic’s equipment, policy, procedures, and conventions. While sharing of the software code between institutions using the same TPS and R&V is certainly feasible, none of the publications have commented on or demonstrated its practicality. One automated checker presented in the literature is both multi‐institutional and multi‐platform, but its functionality is limited to the parsing of documents in portable document format (pdf) and subsequent comparison of plan data consistency between TPS, R&V, and third party applications.^3^, ^9^ Recently, commercial companies have also begun to market and sell automated plan check tools. We were motivated to join a multi‐institutional collaboration to jointly develop a flexible automated checking framework designed with the versatility to be shared across the collaborating facilities while supporting the individual differences between practices. Our plan check tool (PCT) was initially developed at University of Michigan (UM),^10^, ^11^ but later co‐developed between *UM* and *Memorial Sloan Kettering Cancer Center (MSKCC)*.

In this work, we analyze the effect that PCT has had on both the efficiency and effectiveness of initial chart checks at *MSKCC* by comparing identified errors before and after PCT implementation. Our experience co‐developing the software with *UM* and the associated legal, software development, and testing accommodations will be described in a subsequent publication.

## 2. MATERIALS AND METHODS

**Figure 1:**
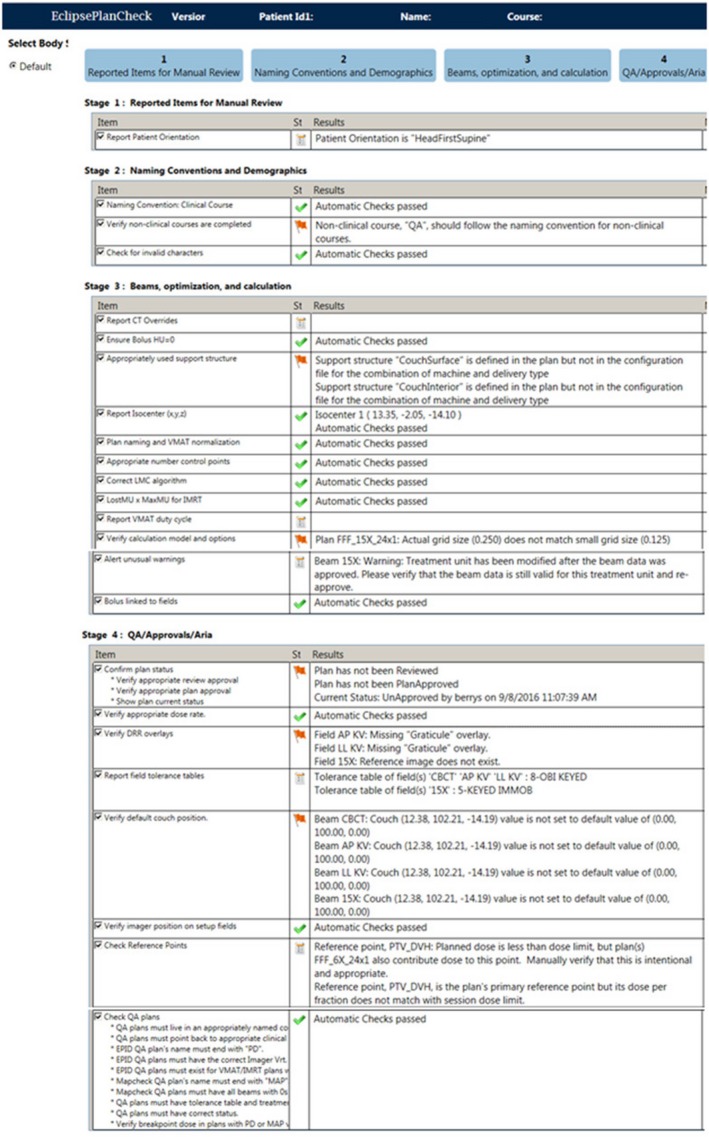
Screen shot of plan check tool output. Each checker listed in the Item column is independent. The order of display, as well as which checkers to use, is configurable. The three output states are as follows: a green checkmark denoting a “pass,” a red flag denoting an item that did not pass the criteria, and a “report” icon, used with checkers that do not check, but only list, information for user review. The Results column gives further information from each check, such as why a flag was thrown.

While PCT is described in more detail elsewhere,^10^, ^11^ in brief, it is a read‐only applications programming interface (API) plug‐in, written in C#, designed for the Eclipse TPS (Varian Medical Systems, Palo Alto, CA). Data elements not available through the API are accessed via SQL queries. The tool consists of three main components: (a) a framework that controls the user interface, initiates database access and loads, (b) individual checkers which either automate a specific check (e.g., isocenter consistency or correct dose calculation algorithm), or serve as a place holder to remind the user to do a manual check, and (c) a configuration file which defines the checkers to be used as well as the configurable parameters that define the passing criteria for each checker (e.g., institution specific beam naming conventions, list of linear accelerator names, dose calculation algorithm name, and default grid size). Each individual checker is independent of the others and can be loaded or not at the discretion of the individual institution. The PCT framework supports four compliance states for each checker: (a) “pass,” indicating that the automated checks passed the criteria defined in the configuration file, (b) “flag,” indicating that the automated checks did not pass the criteria and should therefore be reviewed and potentially corrected by the user, and (c) “report,” applied in situations where the desired outcome is not rigidly defined. The report status is accompanied by a listing of information for user review and action, as appropriate and (d) “manual,” serving as a reminder to the user that they must perform and document this check manually. No “manual” checkers were used in the PCT implementation at our institution. An example of the PCT output is shown in Fig. 1.

At the time of PCT implementation, the checkers fit into two broad categories. The first represented checks of objective technical parameters that constituted important parts of an initial chart check, such as appropriate dose calculation algorithms used, dose grid size correct, couch and imager positions appropriate, and that the quality assurance (QA) plan and clinical plan were consistent with one another. Subjective checks, such as evaluations of plan quality, were avoided in the first implementation as these are more difficult to specify. The second category constituted a check of details not easily remembered by a user, such as items specific to a given treatment unit, for example, which type of couch is installed with a given linac, or compliance with departmental policies, for example, plan and course naming conventions.

To evaluate the impact of the introduction of PCT on initial plan checking in our clinic, data on errors identified during initial chart checks on patient charts containing graphical treatment plans were acquired during two time periods: before the introduction of PCT in the clinic (July 24, 2015 to July 31, 2015) (“pre‐PCT”) and post‐clinical release (April 14, 2016 to May 2, 2016) (“with‐PCT”). Simple calculations with single or parallel opposed fields to a prescription point were not included in the analysis. In the Pre‐PCT phase, the software was not available for use by either the planners or plan checkers. In the with‐PCT phase, the planners were instructed to run the software upon completion of their plan, to resolve any flags, and then to submit the plan to the plan checker. The plan checker would perform an initial chart check by running PCT and supplementing PCT with manual checks of those items in our departmental plan checking procedures that PCT does not check. In both the pre‐PCT and with‐PCT phases, human plan checkers were asked to record all issues that either they manually detected or that were detected by PCT while completing their initial chart checks, as well as the amount of time, less breaks, or interruptions, it took to check each plan. The version of PCT that was used during the study period consisted of 3 *UM* checkers used without modification, 10 *UM* checkers modified by *MSKCC*, and 21 checkers developed at *MSKCC* (Table 1). Both data collection efforts occurred while using Eclipse version 11.0.47.

**Table 1:** List of the checks that the plan check tool performed at the time of this study.

	Status
UM Checkers being used without modification	
Isocenter the same for all fields	P/F
Report tolerance tables	R
Verify DRR’s created	P/F
UM Checkers modified by MSKCC	
Report patient orientation	R
Image and structure set names follow convention	P/F
Course names follow convention	P/F
Plan names follow convention	P/F
Reference point dose limits	P/F
Bolus attached to all fields	P/F
Overlay on DRR’s	P/F
Dose rate	P/F
Plan status	P/F
Default calculation settings and grid size	P/F
MSKCC Checkers	
Couch structures properly applied	P/F
List unusual warnings	R
Invalid characters	P/F
Valid number of control points	P/F
IMRT leaf delivery	P/F
Correct leaf motion calculator algorithm	P/F
Report CT density overrides	R
Report bolus with non‐zero HU values	R
Non‐clinical courses are set to “complete”	P/F
Default couch position	P/F
Setup fields correct imager position	P/F
QA plan checkers:	P/F
verification plan in QA course	
EPID dosimetry plan naming convention	
EPID reference images at correct SID	
EPID dosimetry if 1 fx, IMRT/VMAT, 6X/15X compare MU/cp against clinical plan	
Mapcheck plan created if single fx and IMRT/VMAT 6X‐FFF	
Mapcheck plans have all beams gantry = 0	
EPID and Mapcheck plans have tolerance tables and time defined	
EPID QA plans should have correct status	
Mapcheck QA plans should have correct status	

“P/F” indicates that the checker outputs a “pass” or “flag” depending on whether or not the item passes the check criteria defined in the configuration file. “R” indicates that the checker just outputs data for the user to review. The last checker (QA plan) includes 10 consititutent checks and will flag if any one does not pass the criteria. Abbreviations: DRR = digitially reconstucted radiograph, FFF = flatttening filter free, fx = fraction, HU = Hounsfield Units, SID = source‐to‐imager distance.

Errors detected by the human plan checkers pre‐PCT and with‐PCT were sorted into 17 categories, 9 un‐related and 8 related to the automated checks performed by PCT. A two‐tailed 2‐by‐2 contingency table using Fisher’s exact test was utilized to determine whether the change in the number of PCT un‐related and related errors from pre‐PCT to with‐PCT was statistically significant. The time per check was recorded pre‐PCT and with‐PCT and were plotted as histograms. The Mann–Whitney *U* test was used to measure whether individual and overall checking times significantly (*P* < 0.05) differed between pre‐PCT and with‐PCT.

## 3. RESULTS

In all, 187 initial chart checks were performed by nine plan checkers in the pre‐PCT period, resulting in 200 issues reported. In total, 150/200 (75%) of the issues were un‐related to checks that would have been performed by PCT and 50/200 (25%) would have been identifiable by PCT had it been clinically available at the time (Table 2). In the with‐PCT period, there was a statistically significant decrease in the fraction of issues recorded by the plan checkers related to checks performed by PCT (*P* < 0.001). Totally, 186 checks recorded by 10 plan checkers resulted in 127 issues reported. Of these, 114/127 (89.76%) were items that the PCT was not designed to catch and 13/127 (10.24%) were items that the PCT would have identified for the planner and therefore should have been fixed before handing the plan into the plan checker. The fact that significantly fewer PCT‐related issues are reported by the plan checkers in the with‐PCT period indicates that that PCT now allows many of these “near‐miss” events to be caught earlier, at the level of the planner. From a quality control perspective, this is preferred since it is one level further removed from the patient.

**Table 2:** The number of checks recorded by the plan checkers both pre‐plan check tool (PCT) and with‐PCT, sorted by category.

Category	# Pre‐PCT	% Pre‐PCT	# with‐PCT	% with‐PCT
Un‐related to checks performed by PCT:				
Missing/Incorrect: contours, Booleans	21	10.5%	7	5.5%
Missing/Incorrect: documentation, billing, database logs	64	32.0%	49	38.6%
Prescription problems	17	8.5%	20	15.7%
Plan quality: DVH, isodose lines unsatisfactory, beam arrangement, missing flash	13	6.5%	5	3.9%
Aria details: treatment time, imaging templates, session scheduling, carepaths, breakpoint	11	5.5%	9	7.1%
DRR quality, window/level	5	2.5%	2	1.6%
Treatment field technical parameters (names, MLC, intensities, jaws, etc.)	7	3.5%	11	8.7%
Setup field technical parameters (angles, names, etc.)	6	3.0%	2	1.6%
Miscellaneous	6	3.0%	9	7.1%
Total:	150	75.00%	114	89.76%
Related to checks performed by PCT:				
Completion status of non‐clinical courses	17	8.5%	3	2.4%
DRR overlays	5	2.5%	1	0.8%
Treatment field technical parameters: bolus, couch coordinates	2	1.0%	0	0.0%
Setup field technical parameters: imager position	2	1.0%	1	0.8%
Reference Points: missing or incorrect contributions	5	2.5%	2	1.6%
Incorrect grid size	3	1.5%	0	0.0%
Course/plan not follow naming conventions	10	5.0%	5	3.9%
Incorrect tolerance tables	6	3.0%	1	0.8%
Total:	50	25.00%	13	10.24%
Grand Total:	200	100.00%	127	100.00%
Number of plan checks:	187		186	

Nine categories are unrelated to checks performed by the plan check tool (PCT) and eight are related to checks performed by PCT. The total number and fraction of issues related to items checked by PCT significantly decreased (*P* < 0.001) after implementation of PCT in the clinic. Abbreviations: DRR = digitally reconstructed radiograph, DVH = dose volume histogram, MLC = multi‐leaf collimator.

Our with‐PCT data indicate that in addition to reducing the problems that PCT can directly identify, there was an overall reduction (64%) in recorded issues of all sorts. This is most probably multi‐factorial and related to the constant evolution of our clinical practice, other departmental safety initiatives, differences in human plan checker compliance with the request to record all issues, and perhaps also PCT allowing the planners to focus their attention on larger issues of plan quality rather than specific technical details. Table 2 shows that the number of plans rejected due to plan quality issues decreased from 13 to 5 and the number of issues related to contours and Booleans decreased from 21 to 7 between pre‐PCT and with‐PCT periods.

**Figure 2:**
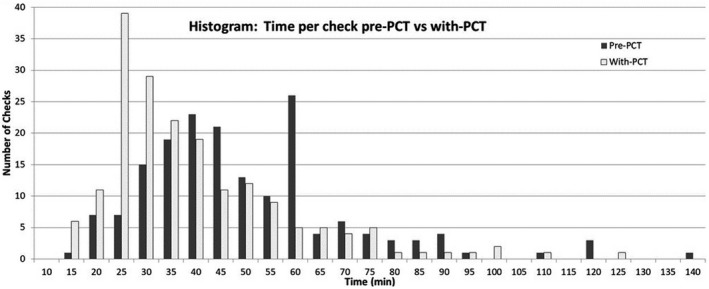
The recorded time, less breaks, and interruptions, per plan check prior to [pre‐plan check tool (PCT)] and after (with‐PCT) the introduction of PCT.

As shown in Table 3, there was also a statistically significant decrease (z‐score = −5.98248, *P* < 0.001) in the overall average (49.4 min vs 39.3 min) and median (45 min vs 35 min) time to perform an initial chart check between the pre‐PCT and with‐PCT time periods using the Mann–Whitney *U* test, although the time savings for individual plan checkers varied. This is also illustrated by the shift of the histogram peak to the left in Fig. 2. This approximate 20% decrease in chart checking time equates to a savings of nearly half a FTE physicist. Although not quantified in this study, this is not expected to indicate that the 10 min saved by plan checkers would be added to the planner's time. Rather, it would also be expected to result in time savings for the planner since they can monitor for and fix problems as they are working on them, prior to plan completion. Under the pre‐PCT QA paradigm at our institution, the initial chart check is typically done the day before the patient starts treatment; sometimes days after the plan was complete. Any issues detected during the plan check are raised with the planner, and the planner is tasked with fixing them, which at that point often means having the planner unlock the plan, fix the problems, and seek out the physician for re‐review. Therefore, detection of problems during the planning process itself should improve the efficiency of the planning process for all team members.

**Table 3:** The time per check, both pre‐plan check tool (PCT) and with‐PCT, for 10 plan checkers.

Plan checker	# Checks pre‐PCT	Time		# Checks with‐PCT	Time		Difference in average	Difference in median	Mann‐Whitney U test *P*‐value
		Average (min)	Median (min)		Average (min)	Median (min)			
A	28	48	40	29	30.7	25	0.64	0.63	**<0.001**
B	22	60.3	51	22	43	41.5	0.71	0.81	**0.026**
C	19	42.1	40	20	38.25	35	0.91	0.88	**0.032**
D	13	50.5	45	32	38.2	35	0.76	0.78	**0.018**
E	21	64.3	60	15	54.6	52.5	0.85	0.88	0.263
F	30	36.9	37.5	22	26	21	0.70	0.56	**0.010**
G	2	39	39	14	36.1	31.5	0.93	0.81	N/A
H	24	48.3	45	17	50	39	1.04	0.87	0.575
I	28	58.2	60	7	42.9	45	0.74	0.75	0.055
J	0	N/A	N/A	8	40.6	30.5			N/A
Overall	187	49.4	45	186	39.3	35	0.80	0.78	<0.001

The average and median time per check decreased by 20% after implementation of PCT in the clinic. Bold indicates *P*‐value < 0.05.

## 4. DISCUSSION

Prospective collection of pre‐implementation data on identified errors and time spent in the plan checking process have allowed us to measure the impact of a technological innovation, the introduction of PCT, on the safety and workflow of the radiotherapy process at our institution. Furthermore, analysis of this data creates a feedback mechanism whereby future improvements to PCT can be identified.

Five of the 13 with‐PCT near misses that made it to the level of the plan checker despite being items that PCT checked were due to course and plan names not following naming conventions. Prior to PCT implementation, planners were aware of the conventions but the naming was done upon import into Eclipse from the CT simulation scanner software by the radiation therapists, and not usually subsequently modified by the planners, even if non‐compliant with the conventions. Therefore, it was found that the failure to correct this issue, even when flagged by PCT, was due to a lack of user buy‐in on the importance of such naming conventions. Based on this feedback, the checker has been modified to be less stringent while management simultaneously engaged on an education campaign to convince the planners of the importance of naming conventions. One of the 13 near misses was due to the use of an incorrect tolerance table in the R&V system. The PCT tolerance table checker is only partially automated: It will flag for a “non‐clinical” tolerance table (e.g., “TEST”) but as long as the chosen tolerance table can potentially be used clinically the checker will report what is being used for manual inspection and a decision by the user as to whether it is appropriate for that particular plan. In this with‐PCT instance, either the report did not garner the attention of the planner or it did but they misjudged the appropriateness of the choice. This highlights the fact that ideally a checker should only give a pass or a flag, making it clear to the user which items need attention. We intend on introducing this refinement to the tolerance table checker in subsequent versions. The remaining 6 of 13 near misses (5.5% of found issues) were obvious flags that should have been, but were not, fixed by the planner. Their failure to correct them could have been due to a number of possible reasons: neglecting to run PCT at all, running PCT but not noticing, valuing, or understanding the flag output message, or running PCT and appreciating the importance of the flag but not knowing how to fix it. We expect this number to decrease as PCT becomes more incorporated into our departmental culture. User buy‐in and culture are very important in all safety interventions and can be achieved by demonstrating to users that this is both beneficial for the institution (in terms of saved FTE and decreased errors) and beneficial to the individual user (in terms of saving them time and increasing the quality of their output).

Our pre‐PCT data indicate that, at the time of initial implementation, PCT was only able to catch about 25% of the issues that plan checkers encounter. However, it is important to note that with additional development and testing, and the implementation of new PCT checkers, the PCT contribution to error detection is also expected to increase. Between the end of this initial implementation and data collection phase (5/2016) and 10/2019, the number of checkers in use at our institution has increased from 34 to 73. Continual review of near‐miss and error data entered into our institutional incident learning database is very informative in helping us and our collaborators identify priorities for the development of future checkers.

The benefit of establishing a collaboration with a like‐minded institution is that the development and programming work involved with the creation and maintenance of the tool is shared, reducing the resources needed to establish the tool in clinical practice. The benefit of our particular implementation is that the framework, as described in Covington, et al.,^11^ is designed such that each individual checker can be added, removed, re‐configured, or have a modification in the code, without affecting any of the other checkers. This compartmentalization allows for efficiencies in testing and release cycles and enables each institution to benefit from a shared codebase while retaining the ability to individualize the tool to their own institution. Our partner institution also found that the introduction of the tool into their clinic reduced the occurrence of errors, specifically with respect to those related to reference points, naming conventions, setup fields, and bolus. They also realized an efficiency gain, calculating that an estimated 489 h were saved from the automation of 19 checklist items.^11^

A potential shortcoming of our study was that other aspects of our clinical practice could have changed over the 11‐month data acquisition period. For example, there could have possibly been unexpected seasonal variations in the mix of types of patients between acquisition periods in July and April. Also, as shown in Table 3, the number of plans checked by each plan checker varied between the two reporting periods. Although we have a departmental standard plan checking procedure and approach, it is possible that some plan checkers are better at identifying and recording errors than others. We attempted to keep the data collection periods short since the plan checkers were being asked to record this data in the course of their clinical work. A protracted data collection period risked reducing user buy‐in and compliance with recording and reporting results which would have resulted in an undercounting of errors identified, confounding our results.

We report a reduction in all recorded errors during the post‐implementation data collection period, even though many of those errors are not directly related to items that PCT checks. Therefore, it is most likely that other departmental safety initiatives worked in concert with PCT to reduce the total number of errors recorded. For this reason, we analyzed the change in the proportion of errors related to checks carried out by the plan check tool relative to all errors recorded rather than analyzing absolute numbers of errors. Other approaches to quantify the error detection rate of PCT against manual checks could have been performed by retrospectively running PCT on cases that had previously been manually checked. However, while this was done in the context of testing the functionality of PCT, it is not informative with respect to the effect of the implementation of this tool in the clinic as data about time per check would be missing and the same gradual shifts of clinical practice over time would remain confounding to the results. Another option would be to have physicists manually check plans while PCT is simultaneously run on those same cases. However, it would be very challenging to secure enough of the time of clinical experts to acquire sufficient data to be statistically meaningful. Furthermore, having the user cognizant of the head‐to‐head comparison may bias the speed or thoroughness at which they may manually perform the manual check. Therefore, we contend that our method most practically characterizes the influence of PCT at the time of its introduction into our clinical workflow. As the tool grows and evolves, we do intend on continuing to investigate, document, and report on its effect on the efficacy and efficiency of pre‐treatment physics chart checks.

There are many avenues for future development. The first is that newer versions of Eclipse allow writeable scripting, allowing the development of tools to write the expected values for given parameters (e.g., treatment couch coordinates) into the database rather than simply flagging an incorrect parameter for a user to manually adjust. Second, while the initial implementation focused on checks of technical parameters, we are developing automated checks of segmentation and plan quality. Third, since a TPS is just one component of the radiation oncology ecosystem, checks between the TPS and other software, such as segmentation and image registration platforms, institutional electronic medical record databases, institutional scheduling systems, and other departmental software programs, are being considered. Finally, we and our partner institution would potentially be interested in extending the tool to other like‐minded institutions, to further enlarge the pool of development work and the reach of the tool.

## 5. CONCLUSION

We have demonstrated that the use of a multi‐institutional, configurable, automated plan checking tool has resulted in both substantial gains in terms of efficiency increases and moving error detection to earlier points in the planning process, decreasing their likelihood that they reach the patient. The sizeable startup effort needed to create this tool from scratch was mitigated by the sharing, and subsequent co‐development, of software code from a peer institution. Continued growth and maturation of this tool is expected to allow us to continue on our current trajectory of decreasing plan checking time while increasing efficiency.

## CONFLICT OF INTEREST

The plan check tool project is part of a co‐development agreement between MSKCC, University of Michigan, and Varian Medical Systems. Margie Hunt and Sean Berry hold grants from Varian Medical Systems unrelated to this work.
